# Copeptin in anorexia nervosa

**DOI:** 10.1002/brb3.1551

**Published:** 2020-02-19

**Authors:** Jens P. Goetze, René Klinkby Støving

**Affiliations:** ^1^ Department of Clinical Biochemistry, Rigshospitalet University of Copenhagen Copenhagen Denmark; ^2^ Center for Eating Disorders Odense University Hospital Odense Denmark; ^3^ Elite Research Center for Medical Endocrinology Odense University Hospital Odense Denmark; ^4^ Mental Health Services in the Region of Southern Denmark Odense Denmark; ^5^ Clinical Institute University of Southern Denmark Odense Denmark

**Keywords:** anorexia nervosa, antidiuretic hormone, arginine vasopressin, copeptin, insulin

## Abstract

**Objective:**

Antidiuretic hormone (ADH) is involved in the response to stress and in depression and anxiety. However, studies on ADH in anorexia nervosa (AN) show conflicting results. A major reason for this may be methodological challenges due to short half‐life of ADH in circulation and rapid degradation in vitro. To overcome these obstacles, copeptin, the C‐terminal fragment stemming from the ADH precursor, has been increasingly used as a stable clinical measure for ADH. Furthermore, copeptin has been recognized as a biomarker of insulin resistance in obesity.

**Methods:**

We measured fasting copeptin in plasma from 25 normohydrated, stable women with AN (BMI 13.0 ± 2.0) and 25 age‐matched women.

**Results:**

No difference in copeptin levels was found (6.8 ± 1.8 vs. 5.5 ± 0.5 pmol/L). Confirmatory, copeptin concentrations were correlated to insulin resistance assessed by the homeostasis model assessment of insulin resistance.

**Discussion:**

We report for the first time that copeptin level as a marker of ADH activity is not altered in fluid‐ and electrolyte‐stabilized patients with severe AN patients, indicating that ADH may not be crucial in the pathophysiological involvement of psychologic stress in AN.

## Graphical abstract

Antidiuretic hormone (ADH) is involved in response to stress and in depression and anxiety; however, studies in anorexia nervosa (AN) are conflicting, due to short half‐life of ADH in circulation and rapid degradation in vitro. Copeptin has been increasingly used as a stable clinical measure for ADH. We report for the first time that copeptin level is not altered in AN.

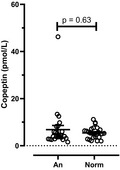

## 1 INTRODUCTION

Antidiuretic hormone (ADH), also referred to as arginine vasopressin, is released from the posterior pituitary to the circulation where it has a number of vital effects on osmoregulation and arteriole constriction, maintaining normal blood pressure and fluid–salt balance. Thus, ADH is released into the bloodstream in response to drop in blood pressure (e.g., orthostatic hypotension) and change in plasma osmotic pressure (e.g., plasma concentration of sodium). However, there are other sources of ADH. For instance, it is synthesized by parvocellular neurosecretory neurons, transported and released at the median eminence, from which it travels through the hypophyseal portal system to the anterior pituitary, where it stimulates corticotropic cells synergistically with corticotropin‐releasing hormone to generate adrenocorticotropic hormone. Different subtypes of ADH receptors have been detected not only in the hypothalamus, but also in the cerebral cortex and the hippocampus where it may be involved in responses to stress, depression, and anxiety (Rutigliano et al., 2016). Depression and anxiety have been reported to be associated with elevated levels of ADH (Csikota et al., 2016) and copeptin (Thomsen et al., 2019) in several different settings such as childhood maltreatment and suicidal behavior.

Anorexia nervosa (AN) is a syndrome with unknown etiology associated with depression, anxiety, and multiple endocrine alterations, which may be adaptive, reactive, or etiologic (Stoving, 2018). The level of ADH has been examined in AN with conflicting results. Partial central diabetes insipidus may occur in patients with active AN with clinical symptoms of polyuria and polydipsia (Kanbur & Katzman, 2011). In ten subjects who were fully recovered from AN, the level of ADH was found to be increased in CSF compared with controls (Frank, Kaye, Altemus, & Greeno, 2000). Conversely, syndrome of inappropriate antidiuretic hormone may also be associated with AN. In a study of 12 patients, it was demonstrated that the osmoregulation was altered both at baseline (normal ADH levels despite lower plasma sodium and osmolality) and after water deprivation (lower urinary concentrating ability). However, this small study population was heterogenic in terms of pharmacologic treatment with antidepressant and oral contraceptives, which are both known to influence ADH secretion (Gilboa et al., 2019).

A major reason for the conflicting results may be the short half‐life of ADH and rapid degradation in vitro. To overcome these methodological challenges, copeptin, the C‐terminal fragment stemming from the ADH precursor, has been increasingly used as a stable clinical measure for vasopressin release (Christ‐Crain, Morgenthaler, & Fenske, 2016). Copeptin measurement in plasma was initially thought of as a sensitive marker in the diagnosis of diabetes insipidus, where the plasma concentrations are markedly reduced. However, copeptin has also been associated with common cardiometabolic disease, namely insulin resistance, diabetes, and heart failure (Alehagen, Dahlstrom, Rehfeld, & Goetze, 2011; Enhorning et al., 2010; Thomsen et al., 2019). Psychological stress is associated with increased levels of cortisol and insulin resistance, and there is evidence that increased ADH secretion may be a link between stress, insulin resistance, and cardiovascular morbidity (Thomsen et al., 2019).

We measured copeptin concentrations in women suffering from severe AN and in age‐matched normal‐weight women. In addition, we tested whether copeptin concentrations in plasma associate with insulin sensitivity, which is a well‐known co‐feature in this particular disorder (Ilyas et al., 2018; Stoving et al., 2007). To the best of our knowledge, this is the first report on copeptin in AN.

## 2 PATIENTS AND METHODS

Samples were collected from a biobank of patients treated in a highly specialized unit for severe AN. The patients fulfilled the diagnostic criteria according the Diagnostic and Statistical Manual of Mental Disorders (DSM) version 5 (American Psychiatric Association (APA) (2013)). The Eating Disorder Inventory‐3 (EDI‐3) profiles are shown in Table S1. All blood samples were obtained between 07:00 and 08:00 in the morning—with the patient in a sedentary position—after a fasting period from 22:00 the previous evening. During the fasting period, the participants did not drink anything, smoke, or chew gum. Patients were fluid‐electrolyte stabilized, and s‐sodium was normal. Serum and plasma were stored at −80°C for later analysis. Twenty‐five women with AN and 25 healthy and age‐matched women were included in the study. The basic features of the cohorts have previously been reported (Goetze, Gustafson, Pedersen, & Støving, 2019). Clinical and biochemical characteristics are shown in Table 1. The study was approved by The Regional Scientific Ethical Committee for Southern Denmark (file no 42053 S‐20140040). Consent was obtained from each patient and subject after full explanation of the purpose and nature of all procedures used.

**Table 1:** Clinical and biochemical characteristic (mean ± *SD*)

	AN (*n* = 25)	C (*n* = 25)	*p*
Body weight (kg)	36.2 ± 7.2	62.7 ± 6.3	<.001
Height (m)	1.67 ± 0.08	1.69 ± 0.06	NS
BMI	13.0 ± 2.0	21.9 ± 1.4	<.001
Subtype Restrictive/Binge or purging	18/7	–	
Disease duration (year)	6 ± 4	–	
Sodium Na (mmol/L)	141 ± 3.8	139.8 ± 4.0	NS
Potassium K (mmol/L)	4.0 ± 0.4	4.0 ± 0.2	NS
Albumin (g/L)	45.8 ± 3.6	45.4 ± 3.0	NS
Creatinine (micromol/L)	62.6 ± 12.6	71.4 ± 11.2	.013
Cholesterol total (mmol/L)	4.4 ± 0.9	4.1 ± 0.8	NS
Triglycerides (mmol/L)	0.9 ± 0.5	0.8 ± 0.4	NS
High‐density lipoprotein cholesterol HDL (mmol/L)	1.7 ± 0.6	1.7 ± 0.4	NS
Low‐density lipoprotein cholesterol LDL (mmol/L)	2.6 ± 0.7	2.3 ± 0.6	NS
Alanine aminotransferase ALT (U/L)	58.4 ± 74.2	17.0 ± 7.2	.008
P‐insulin (pmol/L)	33 ± 25	65 ± 38	.001
Glu (mmol/L)	4.2 ± 1.2	4.9 ± 0.4	<.001
HOMA‐IR^a^	1.1 ± 0.9	2.4 ± 1.7	.002

a HOMA‐IR: The homeostasis model assessment of insulin resistance calculated as [glucose in molar units]/([insulin in picomol unit] * 22.5).

Sodium, potassium, albumin, and creatinine were measured by enzymatic assays on a Roche/Hitachi cobas c system. Serum total cholesterol, high‐density lipoprotein cholesterol, and low‐density lipoprotein cholesterol were determined using the phosphotungstic acid magnesium chloride precipitation method. Serum immunoreactive insulin levels were measured using an enzyme‐linked immunosorbent assay. Alanine aminotransferase (ALT) was analyzed by enzymatic colometric method with pyridoxal phosphate activation. Plasma copeptin was analyzed with an automated immunofluorescence assay on a KRYPTOR platform (ThermoFischer, Henningsdorf, Germany). Specifications of the copeptin assay were as follows: detection limit 0.9 pmol/L, intra‐assay coefficient of variation (CV) < 15%, and inter‐assay CV < 17%.

## 3 RESULTS

**Figure 1:**
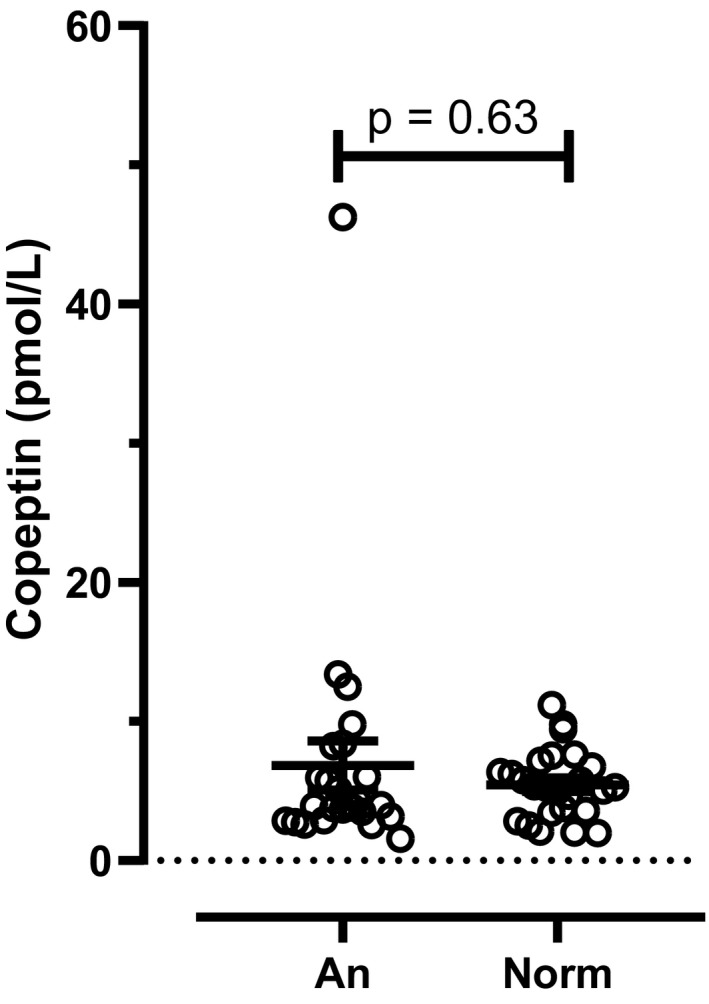
The measured copeptin concentrations in the two groups (*n* = 25 in each group)

First, we examined the difference in copeptin concentrations between groups (Figure 1). Mean concentrations were 6.8 ± 1.8 versus 5.5 ± 0.5 pmol/L, respectively. No difference was found using Mann–Whitney statistics (*p* = .63). Second, we compared copeptin concentrations in both groups against the homeostasis model assessment of insulin resistance (HOMA‐IR). HOMA‐IR differed between the groups (1.09 ± 0.19 vs. 2.40 ± 0.33, *p* < .0001). In order to address metabolic status regression analyses for copeptin against HOMA‐IR, plasma insulin and plasma glucose concentrations were performed, and all correlations were found to be significant (goodness of fit between 0.32 and 0.46, *p* < .05 for all).

## 4 DISCUSSION

Our data show that copeptin, a stable marker of ADH in plasma, is remarkably unchanged in normohydrated stable AN patients with extremely low BMI. Copeptin is suggested to be a more reliable biomarker for the stress level than cortisol, due to its higher stability (Lukaszyk & Malyszko, 2015). To adjust for obvious short‐term effects of dehydration, hyponatremia, and excessive physical exercise, which are common features in AN, the samples were collected during hospitalization after fluid and electrolyte stabilization, and with no exercise in the preceding 24 hr. If we had not stabilized first, a potential difference from the healthy controls would have been hard to interpret. However, an increased level of copeptin was still to be expected due to the extremely low BMI and to anxiety and depression levels associated with AN. Thus, our findings do not directly confirm copeptin as a suitable biochemical marker for the severity and stress level in AN. However, a potential abnormal response to dehydration, hyponatremia, and excessive physical exercise in AN cannot be excluded from the present study.

Since the publication of the 4th edition of DSM, AN has been subdivided into the restricting and binge/purging types with the latter associated with impulsivity, substance abuse, suicide, and depression (Foulon et al., 2007). In our sample, seven of the 25 patients were diagnosed with the subtype binge eating or purging. Purging behavior is strongly associated with dehydration and hyponatremia. However, our study could not detect any difference in copeptin level between patients stabilized during hospitalization and the restrictive subtype or the healthy normal‐weight women.

In accordance with previous studies (Ilyas et al., 2018), we found highly significant increased insulin sensitivity in AN. This is of interest because the copeptin level has repeatedly been reported to be a marker of insulin resistance (Saleem et al., 2009). Thus, AN appears as a condition characterized by endocrinologic stress and adaptation to starvation with increased insulin sensitivity (Stoving, 2018), coexisting with psychologic stress, anxiety, and depression. The latter has been reported to be associated with increased copeptin levels (Siegenthaler, Walti, Urwyler, Schuetz, & Christ‐Crain, 2014). Thus, speculatively ADH expression and release could be blunted by metabolic adaptive increased insulin sensitivity.

In conclusion, this study reports for the first time that copeptin levels were unaltered in fluid‐ and electrolyte‐corrected women with severe AN, indicating that ADH may not be crucial in the pathophysiological involvement of psychologic stress in AN. Further research is warranted to clarify the potential role of ADH and copeptin in the stress mechanism in AN.

## CONFLICT OF INTEREST

None.

## Data Availability

The data that support the findings of this study are available on request from the corresponding author. The data are not publicly available due to privacy or ethical restrictions.

## References

[brb31551-bib-0001] Alehagen, U. , Dahlstrom, U. , Rehfeld, J. F. , & Goetze, J. P. (2011). Association of copeptin and N‐terminal proBNP concentrations with risk of cardiovascular death in older patients with symptoms of heart failure. JAMA, 305(20), 2088–2095. 10.1001/jama.2011.666 21610241

[brb31551-bib-0002] American Psychiatric Association (APA) (2013). Diagnostic and statistical manual of mental disorders, edn. Washington, DC: APA.

[brb31551-bib-0003] Christ‐Crain, M. , Morgenthaler, N. G. , & Fenske, W. (2016). Copeptin as a biomarker and a diagnostic tool in the evaluation of patients with polyuria‐polydipsia and hyponatremia. Best Practice & Research Clinical Endocrinology & Metabolism, 30(2), 235–247. 10.1016/j.beem.2016.02.003 27156761

[brb31551-bib-0004] Csikota, P. , Fodor, A. , Balázsfi, D. , Pintér, O. , Mizukami, H. , Weger, S. , … Zelena, D. (2016). Vasopressinergic control of stress‐related behavior: Studies in Brattleboro rats. Stress, 19(4), 349–361. 10.1080/10253890.2016.1183117 27187740

[brb31551-bib-0005] Enhörning, S. , Wang, T. J. , Nilsson, P. M. , Almgren, P. , Hedblad, B. O. , Berglund, G. , … Melander, O. (2010). Plasma copeptin and the risk of diabetes mellitus. Circulation, 121(19), 2102–2108. 10.1161/CIRCULATIONAHA.109.909663 20439785 PMC3763235

[brb31551-bib-0006] Foulon, C. , Guelfi, J. D. , Kipman, A. , Adès, J. , Romo, L. , Houdeyer, K. , … Gorwood, P. (2007). Switching to the bingeing/purging subtype of anorexia nervosa is frequently associated with suicidal attempts. European Psychiatry, 22(8), 513–519. 17482799 10.1016/j.eurpsy.2007.03.004

[brb31551-bib-0007] Frank, G. K. , Kaye, W. H. , Altemus, M. , & Greeno, C. G. (2000). CSF oxytocin and vasopressin levels after recovery from bulimia nervosa and anorexia nervosa, bulimic subtype. Biological Psychiatry, 48(4), 315–318. 10.1016/S0006-3223(00)00243-2 10960163

[brb31551-bib-0008] Gilboa, M. , Koren, G. , Katz, R. , Melzer‐Cohen, C. , Shalev, V. , & Grossman, E. (2019). Anxiolytic treatment but not anxiety itself causes hyponatremia among anxious patients. Medicine (Baltimore), 98(5), e14334 10.1097/MD.0000000000014334 30702618 PMC6380733

[brb31551-bib-0009] Goetze, J. P. , Gustafson, F. , Pedersen, O. , & Støving, R. K. (2019). MR‐proANP associated with body mass index in patients with anorexia nervosa. The Journal of Applied Laboratory Medicine, 4(1), 132–134. 10.1373/jalm.2018.028290 31639717

[brb31551-bib-0010] Ilyas, A. , Hubel, C. , Stahl, D. , Stadler, M. , Ismail, K. , Breen, G. , … Kan, C. (2018). The metabolic underpinning of eating disorders: A systematic review and meta‐analysis of insulin sensitivity. Molecular and Cellular Endocrinology, 10.1016/j.mce.2018.10.005 30393006

[brb31551-bib-0011] Kanbur, N. , & Katzman, D. K. (2011). Impaired osmoregulation in anorexia nervosa: Review of the literature. Pediatric Endocrinology Reviews, 8(3), 218–221. 21525799

[brb31551-bib-0012] Lukaszyk, E. , & Malyszko, J. (2015). Copeptin: Pathophysiology and potential clinical impact. Advances in Medical Sciences, 60(2), 335–341. 10.1016/j.advms.2015.07.002 26233637

[brb31551-bib-0013] Rutigliano, G. , Rocchetti, M. , Paloyelis, Y. , Gilleen, J. , Sardella, A. , Cappucciati, M. , … Fusar‐Poli, P. (2016). Peripheral oxytocin and vasopressin: Biomarkers of psychiatric disorders? A comprehensive systematic review and preliminary meta‐analysis. Psychiatry Research, 241, 207–220. 10.1016/j.psychres.2016.04.117 27183106

[brb31551-bib-0014] Saleem, U. , Khaleghi, M. , Morgenthaler, N. G. , Bergmann, A. , Struck, J. , Mosley, T. H. Jr , & Kullo, I. J. (2009). Plasma carboxy‐terminal provasopressin (copeptin): A novel marker of insulin resistance and metabolic syndrome. Journal of Clinical Endocrinology and Metabolism, 94(7), 2558–2564. 10.1210/jc.2008-2278 19366852 PMC2708945

[brb31551-bib-0015] Siegenthaler, J. , Walti, C. , Urwyler, S. A. , Schuetz, P. , & Christ‐Crain, M. (2014). Copeptin concentrations during psychological stress: The PsyCo study. European Journal of Endocrinology, 171(6), 737–742. 10.1530/EJE-14-0405 25249697

[brb31551-bib-0016] Stoving, R. K. (2018). MECHANISMS IN ENDOCRINOLOGY: Anorexia nervosa and endocrinology: A clinical update. European Journal of Endocrinology, 180(6), R9–R27. 10.1530/EJE-18-0596 PMC6347284 30400050

[brb31551-bib-0017] Stoving, R. K. , Chen, J.‐W. , Glintborg, D. , Brixen, K. , Flyvbjerg, A. , Horder, K. , & Frystyk, J. (2007). Bioactive insulin‐like growth factor (IGF) I and IGF‐binding protein‐1 in anorexia nervosa. Journal of Clinical Endocrinology and Metabolism, 92(6), 2323–2329. 10.1210/jc.2006-1926 17389700

[brb31551-bib-0018] Thomsen, C. F. , Dreier, R. , Goharian, T. S. , Goetze, J. P. , Andersen, L. B. , Faber, J. , … Jeppesen, J. L. (2019). Association of copeptin, a surrogate marker for arginine vasopressin secretion, with insulin resistance: Influence of adolescence and psychological stress. Peptides, 115, 8–14. 10.1016/j.peptides.2019.02.005 30779927

