# Supplementary material for: Copeptin in anorexia nervosa
Source: Brain Behav. 2020 Feb 19;10(4):e01551. doi: 10.1002/brb3.1551 (PMC7177552; doi:10.1002/brb3.1551)
Supplement: Supplementary file 1 [file BRB3-10-e01551-s001.pdf]

Supplementary Table 1. Eating Disorder Inventory (EDI)-3.

	**AN (n= 25)**
Drive for Thinnes	15.4 ± 7.4
Bulimia	6.1 ± 7.3
Body Dissatisfaction	25.6 ± 8.3
Low Self-Esteem	14.8 ± 6.6
Personal Alienation	13.4 ± 6.7
Interpersonal Insecurity	10.7 ± 5.8
Interpersonal Alienation	9.3 ± 5.7
Interoceptive Deficits	17.1 ± 10.0
Emotional Dysregulation	8.8 ± 6.8
Perfectionism	10.6 ± 6.4
Ascetism	11.4 ± 6.9
Maturity Fears	12.3 ± 6.9
